# Observations on the Effects of Dr. Fowler's Mineral Solution in Lepra and Other Diseases

**Published:** 1806-04

**Authors:** Thomas Girdlestone

**Affiliations:** Physician at Yarmouth


### Medical and Phyfical Journal.

VOL. XV.] APRIL, 1806. [NO. 86.

*Printed for R. PHILLIPS, by W. Thorne, Red Lion Court, Fleet Street, London.*

### OBSERVATIONS ON THE EFFECTS OF DR. FOWLER'S MINERAL SOLUTION IN LEPRA AND OTHER DISEASES; *communicated in a Letter to a Physician in London, by* THOMAS GIRDLESTONE, M. D. *Physician at Yarmouth.*

SIR,

ALTHOUGH I had frequently used, with success, the arseniate of pot-ash in mesenteric and many other scrofulous affections, yet your old acquaintance Mr. B. was the first patient on whom I tried the effects of this medicine in lepra. You remember that he had laboured for fourteen years under that disease, tried repeated salivations, many physicians, and every quack medicine, without any effect, and was at last cured by small and repeated doses of the arseniate of potash. It is now several years since his cure was completed, and my experiments and success have extended to some hundreds of cases of lepra, lichen, prurigo, psoriasis, tinea capitis, &c. &c.

My sesond case of lepra proved very instructive to me. The patient had tried different physicians and quack medicines, with but little palliation of symptoms. After three doses of eight drops, in the twenty-four hours, a lobster redness came over the whole skin, and his face exhibited the commencement of an erysipelatous inflammation. I desired him to take some senna tea to relieve his bowels, and to wait till this redness should subside, and to begin with the solution in doses of half the number of drops. In about two days he was able to resume his medicine, and under the diminished doses, his disease gradually disappeared.

The termination of my third case proved equally instructive; for, on the disappearance of the lepra, there came upon the nates large biles, which gradually dried up, and went away.

I afterwards found that the declination of cutaneous diseases, from this medicine, was marked sometimes by an increase of the eruption, sometimes by large biles, or fissures, about the feet, toes, hands or fingers. One patient, who had laboured for two years under a large patch of the lepra nigricans on his cheek, which had resisted the efforts of different practitioners, was ordered by me four drops of the solutio mineralis twice a day. But the first dose produced the lobster redness of the skin, a distention of the abdomen, and slight sickness. He was relieved by a grain of calomel, and his lepra was afterwards cured by two drops of the solution, taken twice a day for six weeks. On a slight return of the disease, he resumed the medicine in a dose of four drops, which produced the same unpleasant effects as when he tried it before in that dose; but he succeeded in his cure by the use of two drops twice a day, without any inconveniency.

The largest doses which I ever gave of this medicine were twelve drops three times in a day; but I afterwards found that half that dose would equally soon succeed in most cases. The insipidity of the solution, by itself, having tempted some to proceed beyond the doses which I had directed, I soon found it necessary to combine this medicine with an equal portion of tincture of cascarilla, gentian, or cinnamon, and to caution, in the most particular manner, the patient from increasing the drops beyond a certain number. For though I have sometimes succeeded, in a few days, in lessening the severity of cutaneous diseases, yet my experience has taught me not to look for a cure with any dose in less than six or seven weeks. A gentleman, in a distant county, was given by a surgeon twenty drops of the solutio mineralis three times a day, for more than three months, before his lepra was taken away. The doses appear to me to have been far too large, as it had established a weakness on his bowels, and did not take away his lepra so soon, by four or five weeks, as the smaller doses of this medicine generally do. This gentleman was afterwards troubled with nervous symptoms and giddiness of the head, which had resisted the efforts of two physicians in the country, and four in London, before he become my patient. Small doses of calomel and opium, and of ferrum ammoniacum, varied according to symptoms, and persisted in for some weeks, took away the diarrhœa, giddiness, and nervous feelings, and re-established his health.

I have

I have stated the worst symptoms which have arisen from this medicine, under my own directions; but that sickness, pain of the abdomen, nasal hæmorrhage, cough, icteric symptoms, and dropsy, may be induced by it, I have too many opportunities of observing from the solution unfortunately having become a very common medicine with unprofessional people, in curing the intermitents of children.

I saw one child who lost his nails, hair, and part of his skin from this solution, which a lady had given him in improper doses.

From all that I have observed of the abuse of the arsenical solution, I am inclined to attribute the cough, when it arises, to the effects of this medicine on the liver. Too large doses of it generally gives an icteric colour to the urine; and calomel, with or without opium, I have found the best antidote.

In those unfortunate venereal cases, where mercury at first relieves, and very soon after aggravates the ulcerations, I have seen the arsenical solution, with small doses of opium, prevent the extension of the ulcers, where the nitric acid, muriate of lime, sarsaparilla, and other boasted remedies had no power. Under the observation of a cautious practitioner, mercury and arsenic may be made alternately* to assist each other in the cure of many diseases. So far from arsenic inducing consumption, when administered in proper doses, I can produce the evidence of the remains of a consumptive family, who owe their existence, for these last seven or eight years, to the arsenic, which Dr. Beddoes gave them. One of the sisters had died in this county, and the other at Bristol; and the rest were labouring under the same sort of mesenteric affections, as preceded the symptoms of phthisis pulmonalis of those who died, when Dr. Beddoes *gave* the arsenical solution, and restored them to health.

Though I have generally succeeded in curing many cutaneous diseases, and most cases of lepra, yet in some the disease has returned. It has, however, generally returned in a slighter degree, and yielded to a similar course of treatment. I have invariably found the truth of Dr.

X 2 Falconer's

* By *alternately*, in the above sentence, I mean, not that each medicine should be given by turns, through the same days; but that when one medicine has been tried for some days or weeks, the other may be substituted, and persisted in, till the first may again become necessary.

Falconer's remark, that the lepra is first brought on by the sudden application of cold to the body when it is in a heated state. And to the same cause, I believe, may be traced many of the relapses. The cautions which I think necessary in the administration of the arseniate of potash are these.

To begin with it always in the smallest doses, and never to increase the dose beyond five or six drops three times a day; and to persist only in such doses as can be taken without any inconveniency. In children, the dose should only be from one to two, three, or four drops, once or twice in a day.

The safest method of giving this medicine is in drops; and in order to prevent it from becoming a common, and consequently a disgraced medicine, it is necessary to combine it with an equal portion of some harmless tincture, as of cascarilla, gentian, or cinnamon, &c. and the drops may be ordered to be taken in a decoction of sarsaparilla or mixture with tincture of cardamoms, or any other spicy tincture.

In cutaneous diseases some patients are too costive, and some are too much inclined to purge. The arsenical solution alone is sometimes sufficient to regulate the bowels; where that is not the case some addition may be necessary. To the costive, calomel may become necessary. To those whose bowels are too much relaxed, the fourth of a grain of opium, or less, given once, twice, or thrice in a day, may assist the solution.

Where the itching of the skin is so very troublesome, hydrarg. muriat. dissolved in aqua rosæ, or aqua calcis, will often afford relief. And in the prurigo senilis, or herpes senilis, the washing the skin every night and morning with warm water, and the strict attention to an anti-dyspeptic diet greatly assist the medicine.

I have for some time used the solutio mineralis in cases of tape-worm. That this medicine, with the use of purgatives, brings away larger portions than any purgative medicine does without it, my present experience leads me to believe. And I have found the solutio mineralis a most powerful destroyer of the ascaris lumbricoides. It requires so many months to be satisfied that the tape-worm is killed, that a longer time must pass away before I am able fully to appreciate the powers of arsenic over this disease. But as the tænia osculis marginalibus appears to be an indigenous disease, in and about Yarmouth, I have a good opportunity of extending my experiments. My servants servants once found this worm in a rabbit, and once in an eel; in dogs it is very common. We have lakes about Yarmouth, but I find as many of the inhabitants who use pump water, as those who use the water of the open wells, labour under this disease,

I am, &c.

*Yarmouth, Feb.* 20, 1806. THOMAS GIRDLESTONE.

